# 

**Published:** 1888-10

**Authors:** 


					﻿OCTOBER, 1888.
OLD SERIES
Vot XXV

NEW 8^^
Vol.
K<
4DDffl«S
’^W.F.WESTMORELAND.M dJW
H.V.M.MILLER.M.D.LL.D^^
•y '"'	"”'
^bushed Br JAMES PiiARRISQN&Cgi
t_________ ■gTLANTA.GA.
suBsornrrioNi t2.eo year.
				

